# Effects of Dapagliflozin on 24-Hour Glycemic Control in Patients with Type 2 Diabetes: A Randomized Controlled Trial

**DOI:** 10.1089/dia.2018.0052

**Published:** 2018-10-25

**Authors:** Robert R. Henry, Poul Strange, Rong Zhou, Jeremy Pettus, Leon Shi, Sergey B. Zhuplatov, Traci Mansfield, David Klein, Arie Katz

**Affiliations:** ^1^ Division of Endocrinology and Metabolism, University of California San Diego School of Medicine, San Diego, California.; ^2^ Center for Metabolic Research, VA San Diego Healthcare System, San Diego, California.; ^3^ Integrated Medical Development, LLC, Princeton Junction, New Jersey.; ^4^ Medpace, Inc., Cincinnati, Ohio.; ^5^ AstraZeneca, Fort Washington, Pennsylvania.

**Keywords:** Continuous glucose monitoring, Daily glycemic variability, Dapagliflozin, SGLT2 inhibitor

## Abstract

***Background:*** Glycated hemoglobin (HbA1c) and measures of short-term glycemia do not fully capture daily patterns in plasma glucose dynamics. This study evaluated 24-h glycemic profiles in patients with type 2 diabetes (T2D) initiated on dapagliflozin treatment using continuous glucose monitoring (CGM).

***Methods:*** This randomized double-blind placebo-controlled multicenter parallel-design 4-week study compared dapagliflozin (10 mg/d; *n* = 50) with placebo (*n* = 50) in adult patients with T2D uncontrolled (HbA1c 7.5%–10.5%) on either stable doses of metformin monotherapy (≥1500 mg/d) or insulin (≥30 U/d with or without up to two oral antidiabetes drugs). CGM was used to measure 24-h glycemic profiles for 7 days pretreatment and during week 4 of treatment. The primary outcome was change from baseline in 24-h mean glucose (MG) at week 4.

***Results:*** The 24-h MG decreased 18.2 mg/dL with dapagliflozin and increased 5.8 mg/dL with placebo (*P* < 0.001). The proportion of time spent in the target glucose range (70–180 mg/dL) increased significantly with dapagliflozin versus placebo (69.6% vs. 52.9%; *P* < 0.001), with a small (0.3%) increase in time spent in the hypoglycemic range (<70 mg/dL), driven by those on background insulin therapy. Dapagliflozin reduced postprandial glucose and significantly decreased overall glucose variability. Few events of symptomatic hypoglycemia occurred. The most common adverse event was urinary tract infection (6% in each treatment arm).

***Conclusions:*** Compared with placebo, dapagliflozin improved measures of glycemic control and variability as assessed by CGM. Glycemic improvements were more pronounced in the group on background metformin than those receiving basal insulin.

## Introduction

Glycated hemoglobin (HbA1c), which reflects average overall glycemia for a period of ∼3 months, is a well-validated surrogate for the microvascular complications of diabetes mellitus.^1^ Although HbA1c and other measures of more short-term glycemia are useful for monitoring patients' responses to treatment over time, they do not capture daily patterns in plasma glucose dynamics and are poor predictors of hypoglycemia.^2,3^ Other glycemia measures, such as fasting plasma glucose (FPG), may also fall short of assessing glycemic control.^4^

Continuous glucose monitoring (CGM) can provide information about glucose dynamics not gleaned from static glucose measurements. The use of CGM among patients has been shown to reduce time spent outside of glucose targets for a 24-h period, lower HbA1c, and more accurately identify hypoglycemic episodes,^5–7^ thereby helping to characterize hypoglycemia risk.^7,8^

Dapagliflozin is a highly selective reversible inhibitor of sodium–glucose cotransporter 2 (SGLT2) approved for the treatment of type 2 diabetes (T2D). By inhibiting SGLT2, dapagliflozin increases urinary glucose excretion, thereby lowering blood glucose concentrations. Once-daily oral administration of dapagliflozin results in rapid and sustained glucosuria for 24 h in patients with T2D, inhibiting up to 40% of filtered glucose from reabsorption by the kidneys, for a glucose excretion rate of up to 3 g/h (≈70 g/d).^9^ The pharmacodynamic properties of dapagliflozin have translated into consistent improvements in glycemic control in a wide spectrum of patients with T2D, both as monotherapy and in combination with other oral antidiabetes drugs (OADs) and/or insulin.^10^

The objective of this study was to compare the effects of dapagliflozin versus placebo on the 24-h glycemic profile assessed by CGM in adult patients with T2D uncontrolled on stable doses of either metformin alone or insulin (with or without up to two OADs).

## Materials and Methods

### Study design

This was a randomized double-blind placebo-controlled multicenter parallel-design study (ClinicalTrials.gov identifier: NCT02429258) conducted at 34 centers in the United States between May and October 2015. Supplementary Figure S1 (Supplementary Data are available online at http://online.liebertpub.com/suppl/doi/10.1089/dia.2018.0052) depicts the study design. An equal number of patients were recruited to each stratum (insulin or metformin) before randomization. Randomization to dapagliflozin or placebo was performed using an Interactive Voice Response System/Interactive Web Response System in a 1:1 ratio; metformin or insulin background therapy was used as stratum in the randomization.

The Dexcom G4^®^ PLATINUM CGM system (Dexcom, Inc., San Diego, CA) was used to measure patients' interstitial glucose concentrations. A 7-day assessment using the CGM system was performed during the lead-in period (days −14 to −7) and during week 4 of treatment (days 21 to 28). The CGM system recorded data every 5 min (288 times per day). Both patients and investigators were blinded to CGM readings. Patients were instructed to calibrate the CGM device by recording a self-monitored blood glucose value minimally every 12 h according to the manufacturer's instructions.

### Participants

Adult patients (aged ≥18 to ≤75 years) with T2D were eligible if they had been treated with either a stable dose of metformin alone (≥1500 mg/d) or a stable dose of insulin (≥30 U/d with or without up to two OADs) for at least 8 weeks before screening.

At screening, patients were required to have a HbA1c ≥7.5% and ≤10.5%, body mass index (BMI) ≤45 kg/m^2^, and fasting C-peptide concentration ≥1.5 ng/mL. Patients also had to demonstrate ≥70% compliance with the CGM device during the lead-in period, including periodic device calibrations and glucose reading verification per the manufacturer's specification.

Patients were excluded if they had a significant cardiovascular history (per investigator's discretion), history of type 1 diabetes or diabetic ketoacidosis within the past 12 months, bariatric surgery or lap-band procedure, bladder cancer, orthostatic hypotension within the past 6 months, uncontrolled hypertension, or ≥2 episodes of severe hypoglycemia during the previous 12 months. Patients were also excluded if they had received any medication known to affect glucose metabolism (e.g., glucocorticoids) or any prescription or over-the-counter weight-loss medications during the previous 3 months.

The institutional review board for each site approved the study design and methods in accordance with the principles defined by the Declaration of Helsinki. All participants provided written informed consent before entering the study.

### Treatment

The study consisted of a 1-week screening period, a 2-week lead-in period, a 4-week treatment period, and a 1-week follow-up period. During the treatment period, patients received oral dapagliflozin once daily (10 mg/d) or placebo for 4 weeks in combination with either open-label metformin extended-release (≥1500 mg/d) or open-label insulin (≥30 U/d with or without up to two open-label OADs).

Patients were instructed to continue their background diabetes medication(s) as previously prescribed for the duration of the study. Any changes in baseline medication doses were at the discretion of the investigator. No insulin diaries or detailed dosing information was collected. Patients in the insulin stratum were allowed to take more than one type of insulin.

Beginning in the lead-in period and continuing throughout the study, patients were asked to follow a standard diabetes weight maintenance diet. Patients were instructed to avoid significant changes to the timing and content of meals during the 7-day CGM assessment period and to refrain from acetaminophen use. On days −13 and 22, patients were provided a standardized breakfast (660 kilocalories, 60% carbohydrate, 15% protein, and 25% fat) and then only water through the completion of the meal test. Postprandial glucose (PPG) was obtained during blood draws for glucose measurements after breakfast (0, 30, 60, 120, 180, and 240 min) during the onsite monitoring visits on these days.

### Outcomes

The primary assessment was change in the 24-h mean glucose (MG) derived from the 7-day CGM data in the second week of the lead-in period to week 4, which was estimated by dividing the area under the 24-h glucose curve (AUC_0–24_) by 24 h and expressed in mg/dL. Average 24-h glucose profiles and percentages of time in low (<70 mg/dL), normal (≥70 to ≤180 mg/dL), and high (>180 mg/dL) glycemic ranges were secondary end points assessing glucose parameters from CGM data. Other secondary end points were 2-h PPG (estimated by dividing the area under the 2-h glucose curve by 2 h from the standardized meal test), HbA1c, FPG, and fructosamine.

Glucose variability end points included mean amplitude of glucose excursion (MAGE), “distance traveled,” and standard deviation (SD) of 24-h glucose. Patients' change in 24-h MAGE was defined as the mean of the absolute difference from nadir to peak for those differences that exceeded the SD of the CGM assessments for 24 h.^11^ The “distance traveled” arc length of the curve for 24 h was approximated using the trapezoid rule from the finite Fourier approximation of glucose concentrations for 24 h of consecutive glucose measurement.^12^

### Safety

The safety population included all patients who received at least one dose of investigational product, analyzed according to the type of agent received. Safety evaluations included the frequency and severity of adverse events (AEs), reported events of hypoglycemia, serum (hematology and clinical chemistry) and urine laboratory tests, electrocardiograms, physical examinations, and vital signs. AEs were classified using the *Medical Dictionary for Regulatory Activities* (version 17.1) system of nomenclature. A treatment-emergent AE (TEAE) was defined as any AE that started on or after the first dose of randomized investigational product or occurred before the first dose and worsened in severity.

### Statistics

The intention-to-treat (ITT) population included all randomized patients who received at least one dose of randomized treatment, and this was used for all efficacy analyses. For the primary end point of 24-h MG, the least-squares mean (LSM), standard errors of LSM, two-sided 95% confidence intervals (CIs) for the mean change within each treatment group, and the LSM difference between treatment groups were calculated. The changes in 24-h MG from baseline to week 4 were analyzed using an analysis of covariance (ANCOVA) model, with change in 24-h MG as the dependent variable, dapagliflozin treatment and metformin/insulin stratum as factors, and baseline 24-h MG as a covariate.

Secondary end points were analyzed using an ANCOVA model similar to the one described for the primary end point. To account for missing data in the analyses of the primary and secondary outcome variables, linear mixed-effects models were used.

The primary end point was tested using α = 0.05. The study had 80% power to detect a 14.5-mg/dL difference in the primary outcome measure for the overall population. Nominal *P* values and/or 95% CIs are presented, where applicable, for the secondary and exploratory end points; however, inferences for treatment differences with respect to these end points should not be made.

Any 24-h CGM profiles with <260 (non-inclusive) data points were censored out, including the half-day profiles on the days when the CGM device was inserted or terminated. CGM profiles with >260 and <288 data points were considered valid, and any missing data points were imputed by linear or cubic spline interpolation.

## Results

### Study disposition

A total of 100 patients were randomized (dapagliflozin, *n* = 50; placebo, *n* = 50) and are included in both the safety and ITT populations (Supplementary Fig. S2). The dapagliflozin group included 23 patients in the metformin stratum and 27 patients in the insulin stratum, and the placebo group included 25 patients each in the metformin and insulin strata. The mean daily metformin dose in the metformin stratum was 1977.1 mg, and the mean daily insulin dose in the insulin stratum was 52.9 units. In the insulin stratum, 28.8% of patients used long-acting insulin alone or with OAD(s). The remaining patients in the insulin stratum used short- or intermediate-acting insulin alone or in combination with long-acting insulin and/or OAD(s) (Supplementary Table S1).

A total of 97 patients with adequate data for both lead-in and week-4 CGM measures completed the study: two patients in the dapagliflozin group withdrew consent and did not complete the study and one patient in the placebo group was withdrawn because of a protocol violation (Supplementary Fig. S2).

### Patient demographics and baseline characteristics

Patient demographics and baseline clinical characteristics were generally similar across treatment groups (Table 1). Patients in the insulin stratum tended to be older, with higher BMI, higher HbA1c, higher systolic blood pressure, and a longer duration of diabetes than patients in the metformin stratum.

**Table 1.** Demographics and Baseline Characteristics of the Study Population

	*Overall (*N* = 100)*		*Metformin stratum (*n* = 48)*		*Insulin stratum (*n* = 52)*	
*Characteristics*	*Dapagliflozin (*n* = 50)*	*Placebo (*n* = 50)*	*Dapagliflozin (*n* = 23)*	*Placebo (*n* = 25)*	*Dapagliflozin (*n* = 27)*	*Placebo (*n* = 25)*
Age at randomization, years, mean (SD)	56.9 (7.1)	56.8 (9.7)	56.0 (6.9)	53.2 (10.7)	57.7 (7.3)	60.4 (7.1)
Gender, *n* (%)						
Male	26 (52.0)	25 (50.0)	13 (56.5)	12 (48.0)	13 (48.1)	13 (52.0)
Female	24 (48.0)	25 (50.0)	10 (43.5)	13 (52.0)	14 (51.9)	12 (48.0)
Race, *n* (%)						
White	39 (78.0)	36 (72.0)	18 (78.3)	18 (72.0)	21 (77.8)	18 (72.0)
Black or African American	11 (22.0)	14 (28.0)	5 (21.7)	7 (28.0)	6 (22.2)	7 (28.0)
Ethnicity, *n* (%)						
Hispanic or Latino	14 (28.0)	16 (32.0)	8 (34.8)	7 (28.0)	6 (22.2)	9 (36.0)
Not Hispanic or Latino	36 (72.0)	34 (68.0)	15 (65.2)	18 (72.0)	21 (77.8)	16 (64.0)
Duration of diabetes, years, mean (SD)	10.5 (6.0)	12.3 (7.4)	6.7 (4.0)	9.5 (6.4)	13.9 (5.6)	15.6 (7.3)
Weight, kg, mean (SD)	96.5 (23.5)	96.0 (20.0)	90.6 (21.2)	93.8 (23.5)	101.6 (24.6)	98.1 (15.9)
BMI, kg/m^2^, mean (SD)	34.3 (5.9)	33.2 (5.6)	32.9 (6.2)	32.0 (5.6)	35.5 (5.5)	34.5 (5.4)
SBP, mmHg, mean (SD)	128.9 (13.5)	127.0 (13.7)	125.7 (11.1)	124.9 (13.6)	131.7 (14.9)	129.1 (13.7)
DBP, mmHg, mean (SD)	77.3 (8.2)	77.3 (9.4)	76.8 (8.5)	76.8 (9.6)	77.8 (9.5)	77.3 (8.2)
Heart rate, beats/min, mean (SD)	71.5 (10.4)	75.2 (10.4)	71.0 (11.1)	73.6 (7.8)	71.9 (9.9)	76.9 (12.5)
eGFR, mL/min/1.73 m^2^, mean (SD)	91.4 (22.0)	89.7 (23.6)	93.6 (17.3)	94.5 (26.2)	89.4 (25.5)	84.8 (20.0)
HbA1c, %, mean (SD)	8.31 (0.79)	8.37 (0.81)	8.02 (0.78)	8.23 (0.86)	8.56 (0.71)	8.51 (0.75)
FPG, mg/dL, mean (SD)	163.6 (69.4)	170.3 (57.0)	155.7 (35.3)	169.4 (51.3)	170.3 (89.0)	171.3 (63.3)
2-h PPG, mg/dL, mean (SD)	223.3 (58.4)	231.5 (46.4)	224.1 (63.7)	231.9 (47.2)	222.6 (54.7)	231.2 (46.6)

BMI, body mass index; DBP, diastolic blood pressure; eGFR, estimated glomerular filtration rate; FPG, fasting plasma glucose; HbA1c, glycated hemoglobin; PPG, postprandial glucose; SBP, systolic blood pressure; SD, standard deviation.

### Comparison of the effects of dapagliflozin on MG within the overall study population

**FIG. 1.**
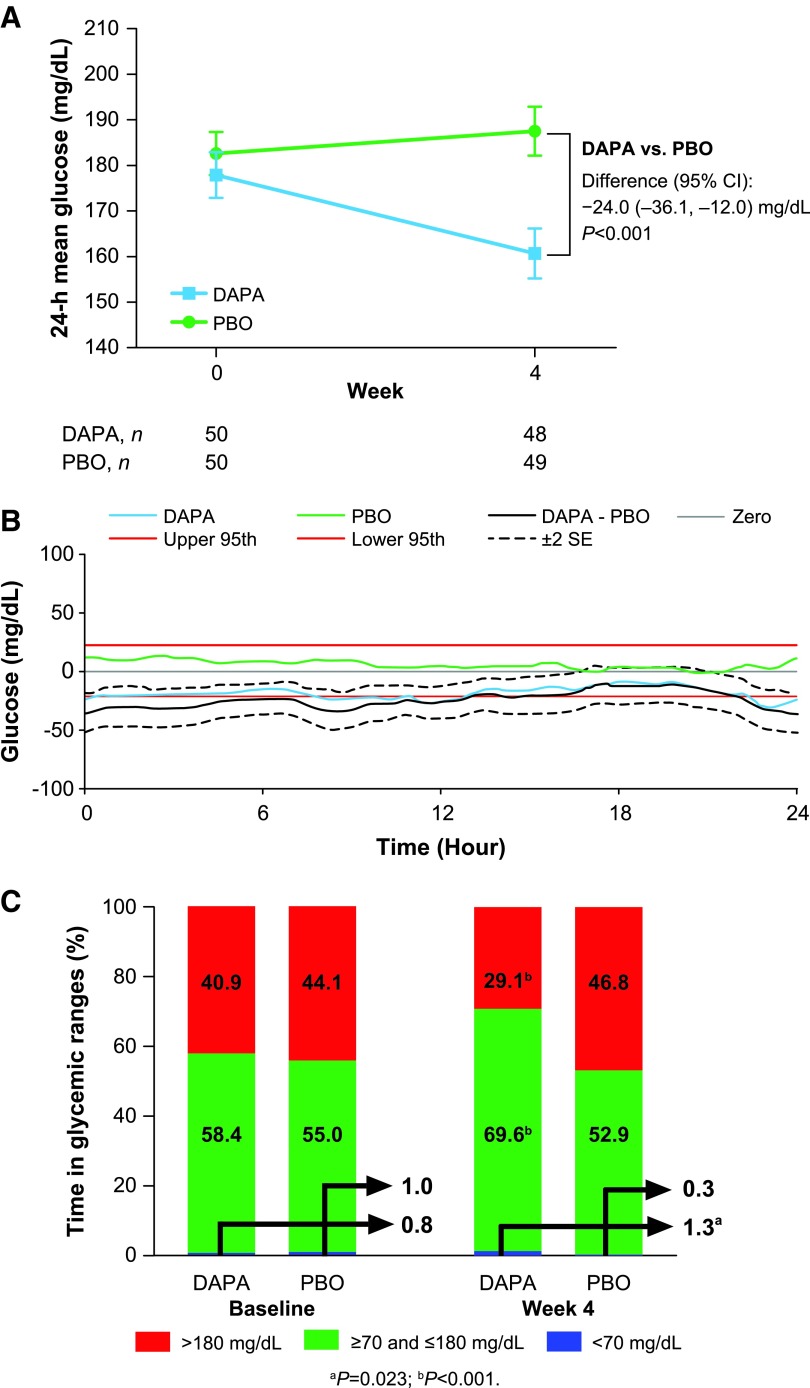
Changes from baseline. **(A)** 24-h mean (SE) glucose, with treatment difference for LSM change from baseline (mg/dL) in the ITT population. **(B)** Comparison of change from baseline in mean 24-h glucose profile at week 4 as shown by MADz in the overall population (time 0 to 24 h means midnight to midnight; the black line represents the treatment DAPA–PBO difference; when the difference between the two groups' change from baseline [blue and green lines for DAPA and PBO, respectively] is outside the MADz red lines [95th percentiles], the two treatments are statistically different at that time of day). **(C)** Change from baseline in time spent (%) in plasma glucose ranges from baseline to week 4 in the ITT population. The arrows denote the percentages of time with glucose <70 mg/dL. CI, confidence interval; DAPA, dapagliflozin; ITT, intention-to-treat; LSM, least-squares mean; MADz, maximum absolute deviation from zero; PBO, placebo; SE, standard error.

In the overall population, 24-h MG decreased in the dapagliflozin group from a baseline of 177.9 to 160.7 mg/dL at week 4 and increased from 182.6 to 187.5 mg/dL in the placebo group (Fig. 1A). The treatment difference for change from baseline between dapagliflozin and placebo in 24-h MG was −24.0 mg/dL (*P* < 0.001). The mean 24-h CGM glucose profile showed a notable downward shift across the overall 24-h profile from baseline to week 4 in the dapagliflozin group, whereas glucose concentrations remained the same or slightly increased across the 24-h profile for patients in the placebo group (Fig. 1B; Supplementary Fig. S3).

Compared with baseline, the mean percentage of time with CGM glucose values within the target range (≥70 and ≤180 mg/dL) increased with dapagliflozin treatment (+12.2%; 95% CI: 7.0 to 17.3), whereas it decreased with placebo (−2.8%; 95% CI: −7.9 to 2.2; Fig. 1C; Supplementary Table S2). The mean percentage of time spent in a hyperglycemic range (glucose >180 mg/dL) at week 4 decreased in the dapagliflozin group (−12.6%; 95% CI: −17.8 to −7.3) and increased in the placebo group (+3.5%; 95% CI: −1.7 to 8.7). The mean percentage of time spent in the hypoglycemic range (<70 mg/dL) at week 4 increased slightly with dapagliflozin (+0.3%; 95% CI: −0.3 to 0.9) and decreased slightly with placebo (−0.6%; 95% CI: −1.2 to −0.1). The mean percentage of time spent with glucose <54 mg/dL was 0.0% at baseline and remained 0.0% at week 4 in both treatment groups.

### Comparison of the effects of dapagliflozin on MG within the individual strata

**FIG. 2.**
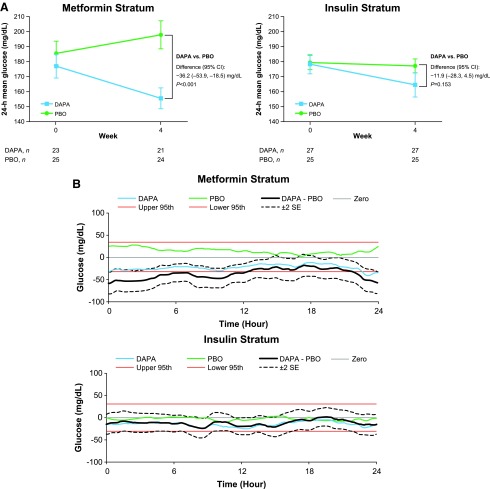
ITT population changes from baseline for the metformin and insulin strata. **(A)** 24-h mean (SE) glucose for the strata, with treatment difference for LSM change from baseline (mg/dL). **(B)** Comparison of change from baseline in mean 24-h glucose profile at week 4 as shown by MADz. CI, confidence interval; DAPA, dapagliflozin; ITT, intention-to-treat; LSM, least-squares mean; MADz, maximum absolute deviation from zero; PBO, placebo; SE, standard error.

The addition of dapagliflozin to metformin reduced 24-h MG from a baseline of 177.0 to 155.5 mg/dL for 4 weeks compared with an increase from 185.6 to 198.0 mg/dL in the placebo group, for an LSM difference of −36.2 mg/dL at week 4 (nominal *P* < 0.001; Fig. 2A). For patients in the insulin stratum, 24-h MG decreased from a baseline of 178.6 to 164.8 mg/dL in the dapagliflozin group and from 179.7 to 177.4 mg/dL in the placebo group; the LSM difference between dapagliflozin and placebo was −11.9 mg/dL at week 4 (nominal *P* = 0.153; Fig. 2A).

Similar to the change in the glucose profile for the overall population, CGM-derived glucose concentrations in the metformin stratum decreased by ∼20 mg/dL across the 24-h profile with dapagliflozin treatment, and slightly increased with placebo (Fig. 2B; Supplementary Fig. S4). In the insulin stratum, glucose concentrations decreased by ∼15 mg/dL with dapagliflozin and decreased slightly with placebo (Fig. 2B; Supplementary Fig. S5).

**FIG. 3.**
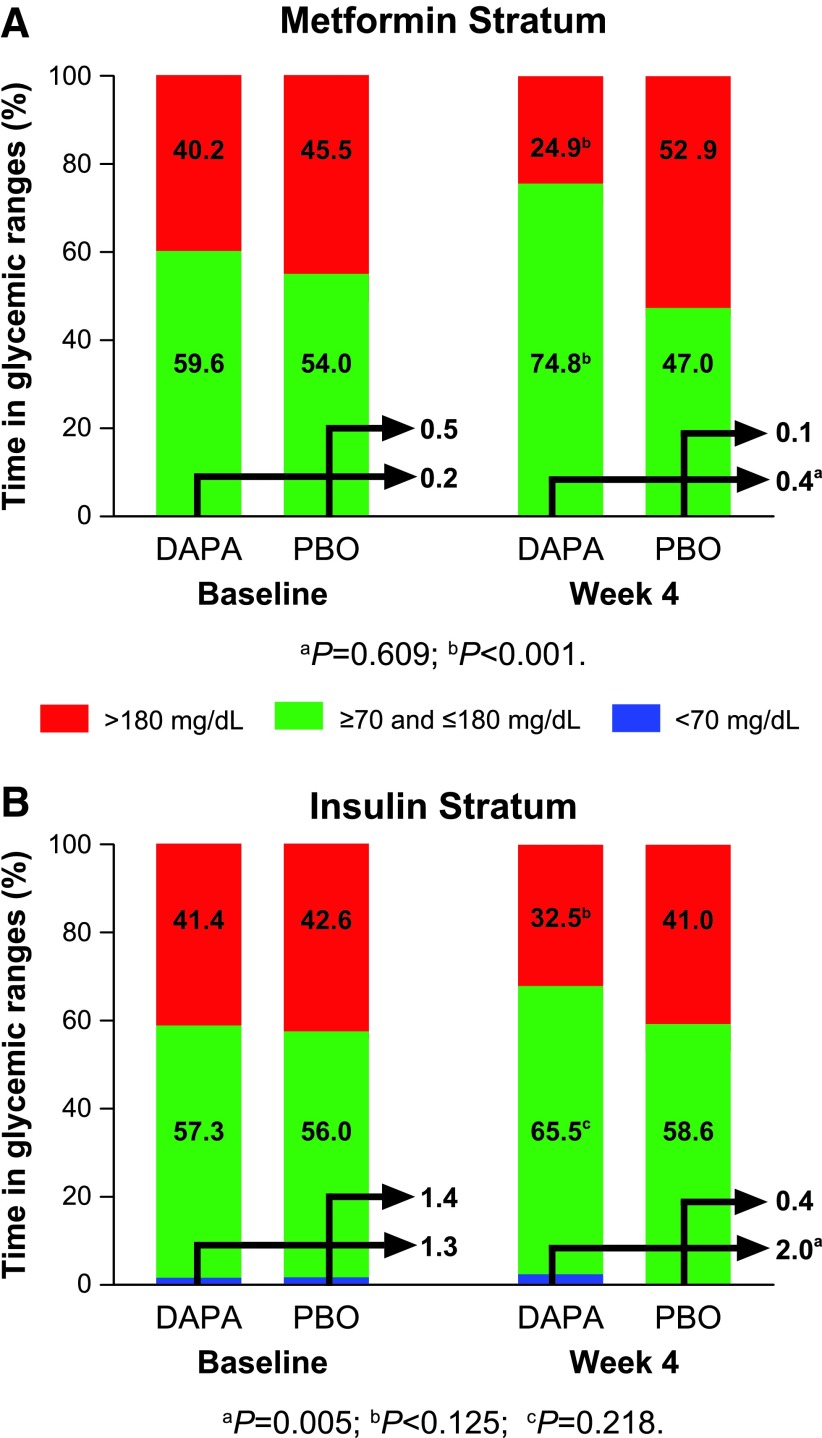
Change from baseline in time spent (%) in plasma glucose ranges from baseline to week 4 in the ITT population. **(A)** Metformin stratum. **(B)** Insulin stratum. The arrows denote the percentages of time with glucose <70 mg/dL. DAPA, dapagliflozin; ITT, intention-to-treat; PBO, placebo.

The increased proportion of time spent in the target range was more pronounced for patients in the metformin stratum (+23.8% vs placebo; nominal *P* < 0.001) than for patients in the insulin stratum (+6.1% vs placebo; nominal *P* = 0.218; Fig. 3; Supplementary Table S2). The mean percentage of time spent in the hypoglycemic range was small in both the dapagliflozin and placebo groups at baseline and week 4 in the metformin and insulin strata. The mean percentage of time spent in the hyperglycemic range from baseline to week 4 decreased with dapagliflozin and increased with placebo in the metformin stratum (Fig. 3A). In the insulin stratum, a decrease was observed in both groups (Fig. 3B).

### Measures of glucose variability

Measures of glucose variability included MAGE, “distance traveled,” and SD of 24-h glucose. For change in MAGE in the overall population, the difference from baseline to week 4 between the dapagliflozin and placebo groups was −15.3 mg/dL (*P* = 0.010; Supplementary Table S3). The difference from baseline to week 4 between dapagliflozin and placebo was −17.7 mg/dL (nominal *P* = 0.040) in the metformin stratum and −12.9 mg/dL (nominal *P* = 0.105) in the insulin stratum.

The mean change in “distance traveled” from baseline to week 4 in the overall population was −28.0 mg/dL in the dapagliflozin group and +9.5 mg/dL in the placebo group, for a difference between groups of −37.5 mg/dL (*P* = 0.312; Supplementary Table S3). The difference between the dapagliflozin and placebo groups at week 4 was −104.1 mg/dL (nominal *P* = 0.057) for the metformin stratum and +29.1 mg/dL (nominal *P* = 0.564) for the insulin stratum.

The mean change in SD of 24-h glucose from baseline to week 4 in the overall population was −3.4 mg/dL in the dapagliflozin group and +1.3 mg/dL in the placebo group, for a difference between groups of–4.7 mg/dL (*P* = 0.037; Supplementary Table S3). The difference between the dapagliflozin and placebo groups at week 4 was −6.8 mg/dL (nominal *P* = 0.041) for the metformin stratum and −2.7 mg/dL (nominal *P* = 0.382) for the insulin stratum.

### Additional glycemic end points

Changes from baseline to week 4 in FPG, HbA1c, and 2-h PPG as measured with blood sampling in the overall population, the metformin stratum, and the insulin stratum are shown in Table 2. A statistically significant decrease from baseline at week 4 with dapagliflozin compared with that of placebo was observed for all three parameters in the overall population and for some parameters in the individual strata. The results in the overall population for 2-h PPG are more similar to those observed in the insulin stratum, whereas those for FPG are more similar to what was observed in the metformin stratum.

**Table 2.** Key Secondary End Points

	*Overall population*		*Metformin stratum*		*Insulin stratum*	
*Secondary end points*	*Dapagliflozin (*n* = 50)*	*Placebo (*n* = 50)*	*Dapagliflozin (*n* = 23)*	*Placebo (*n* = 25)*	*Dapagliflozin (*n* = 27)*	*Placebo (*n* = 25)*
2-h PPG, mg/dL						
Baseline mean (SD)	223.3 (8.3)	231.5 (6.6)	224.1 (13.3)	222.8 (9.4)	222.6 (10.5)	231.2 (9.3)
Adjusted mean (SE) change from baseline to week 4	−49.5 (6.6)	−13.2 (6.5)	−48.9 (8.5)	−7.3 (7.9)	−50.8 (9.6)	−19.0 (9.8)
Adjusted mean (SE) difference vs. placebo	−36.3 (9.3)		−41.6 (11.6)		−31.8 (13.7)	
*P* value for treatment difference	<0.001		<0.001		0.025	
FPG, mg/dL						
Baseline mean (SD)	163.6 (9.8)	170.3 (8.1)	155.7 (7.4)	169.4 (10.3)	170.3 (17.1)	171.3 (12.7)
Adjusted mean (SE) change from baseline to week 4	−26.2 (6.0)	+3.6 (6.0)	−19.2 (7.4)	+10.5 (7.1)	−30.8 (8.7)	−5.5 (9.0)
Adjusted mean (SE) difference vs. placebo	−29.7 (8.5)		−29.8 (10.3)		−25.4 (12.6)	
*P* value for treatment difference	<0.001		0.006		0.049	
HbA1c, %						
Baseline mean (SD)	8.31 (0.11)	8.37 (0.12)	8.02 (0.16)	8.23 (0.17)	8.56 (0.14)	8.51 (0.15)
Adjusted mean (SE) change from baseline to week 4	−0.51 (0.07)	−0.28 (0.07)	−0.41 (0.10)	−0.26 (0.10)	−0.61 (0.10)	−0.31 (0.10)
Adjusted mean (SE) difference vs. placebo	−0.23 (0.10)		−0.16 (0.14)		−0.30 (0.14)	
*P* value for treatment difference	0.024		0.267		0.041	
Coefficient of variation (24-h mean glucose)						
Baseline mean (SD)	0.24 (0.06)	0.24 (0.06)	0.21 (0.05)	0.22 (0.05)	0.27 (0.05)	0.27 (0.06)
Adjusted mean (SE) change from baseline to week 4	0 (0.01)	0 (0.01)	+0.01 (0.01)	+0.01 (0.01)	−0.01 (0.01)	−0.01 (0.01)
Adjusted mean (SE) difference vs. placebo	0 (0.01)		+0.01 (0.02)		0 (0.02)	
*P* value for treatment difference	0.728		0.738		0.880	
HBGI, mg/dL						
Baseline mean (SD)	9.5 (6.6)	10.3 (6.1)	9.0 (7.0)	10.7 (7.5)	10.0 (6.1)	9.9 (4.4)
Adjusted mean (SE) change from baseline to week 4	−2.6 (0.8)	+0.8 (0.8)	−3.2 (1.3)	+2.1 (1.2)	−2.0 (1.1)	−0.6 (1.2)
Adjusted mean (SE) difference vs. placebo	−3.4 (1.2)		−5.4 (1.7)		−1.4 (1.6)	
*P* value for treatment difference	0.005		0.002		0.368	
Time spent in BG 70–140 mg/dL, %						
Baseline mean (SD)	29.8 (19.1)	26.2 (16.7)	29.0 (22.7)	23.4 (18.0)	30.4 (15.9)	28.9 (15.1)
Adjusted mean (SE) change from baseline to week 4	+12.6 (2.8)	−1.7 (2.7)	+16.3 (4.2)	−4.5 (3.9)	+9.0 (3.7)	+1.0 (3.8)
Adjusted mean (SE) difference vs. placebo	+14.4 (3.9)		+20.8 (5.7)		+8.0 (5.3)	
*P* value for treatment difference	<0.001		<0.001		0.137	

Study was only powered for the overall population; although *P* values are supplied for the individual strata, inferences for treatment differences should not be made.

BG, blood glucose; FPG, fasting plasma glucose; HbA1c, glycated hemoglobin; HBGI, high blood glucose index; PPG, postprandial glucose; SD, standard deviation; SE, standard error.

### Safety evaluation

Overall, 28 patients (28.0%) had a TEAE during the study (dapagliflozin, *n* = 18 [36.0%]; placebo, *n* = 10 [20.0%]; Table 3). Most TEAEs occurred in one patient per treatment group (Supplementary Table S4). The most common AE was urinary tract infection, reported in three patients (6%) in each of the dapagliflozin and placebo groups (Table 3). AEs were generally evenly distributed between the metformin and insulin strata. The majority of TEAEs were considered mild (71.4%) or moderate (25.0%). One patient in the dapagliflozin group had a severe TEAE of elevated blood creatinine and decreased glomerular filtration rate, which did not result in discontinuation.

**Table 3.** Overview of Adverse Events, Including Treatment-Emergent Adverse Events in ≥2 Patients: Safety Population

*Category,* n *(%)*	*Dapagliflozin (*n* = 50)*	*Placebo (*n* = 50)*	*Overall (*N* = 100)*
Patients with any AE	21 (42)	14 (28)	35 (35)
Patients with any TEAE	18 (36)	10 (20)	28 (28)
Urinary tract infection	3 (6)	3 (6)	6 (6)
Upper respiratory tract infection	1 (2)	2 (4)	3 (3)
Anemia	1 (2)	1 (2)	2 (2)
Oropharyngeal pain	2 (4)	0	2 (2)
Patients with any SAE	1 (2)	0	1 (1)
Patients with any AE leading to discontinuation of drug	2 (4)	0	2 (2)
Deaths	0	0	0

Patients with multiple events in the same category were counted only once in that category. Patients with events in more than one category were counted once in each of those categories.

Safety population consisted of all patients who received at least one dose of treatment, analyzed according to the type of investigational product received.

AE, adverse event; SAE, serious adverse event; TEAE, treatment-emergent adverse event.

One patient in the dapagliflozin group experienced a serious AE of worsening urinary tract infection before initiating study treatment with dapagliflozin; the serious AE resolved, and the patient continued into the study. Overall, two patients had an AE leading to discontinuation of study drug (both in the dapagliflozin group): one patient experienced abdominal discomfort, flatulence, and headache starting on study day 1, and the other patient had fungal genital infection starting on day 27.

Six patients (three in each of the dapagliflozin and placebo groups) in the insulin stratum had documented symptomatic or probable symptomatic hypoglycemic events, all of which were considered nonsevere. Two patients (both in the dapagliflozin group: one patient in the insulin stratum and the other patient in the metformin stratum) had nonsevere asymptomatic hypoglycemic events.

Overall, no clinically meaningful changes from baseline were observed in clinical laboratory tests, vital signs, or physical examination; however, decreases in weight and blood pressure were observed for patients treated with dapagliflozin. From baseline to week 4, mean weight decreased from 96.5 to 94.9 kg, respectively, mean systolic blood pressure decreased from 128.9 to 126.4 mmHg, and mean diastolic blood pressure decreased from 77.3 to 76.2 mmHg.

## Discussion

This is one of the first studies, and the largest, to use CGM to assess the effects of the SGLT2 inhibitor dapagliflozin on the 24-h glucose profile and daily glucose variability among patients with T2D. Guidelines recommend metformin monotherapy as first-line treatment, with the rapid (after 3 months) addition of a second or third antidiabetes agent and/or insulin if glycemic goals are not met.^13^ A large retrospective study showed that ∼85% of patients with T2D in primary care are prescribed metformin and almost 25% receive insulin.^14^ Thus, the patients included in this study, on background metformin or insulin (plus up to two OADs), represent the diverse population of patients with T2D commonly encountered in clinical practice.

The results of this study showed significant improvements in 24-h glycemic control among patients on stable doses of metformin alone or insulin (with or without up to two OADs) treated with dapagliflozin. In the overall population, treatment with dapagliflozin resulted in significant reductions in the primary end point of 24-h MG relative to placebo after 4 weeks of treatment, as well as significant increases in time spent in the target range (≥70 and ≤180 mg/dL), improvements in glycemic parameters (FPG, 2-h PPG, HbA1c, and fructosamine), and glucose variability as assessed by MAGE.

When the results were examined by background medication stratum (metformin vs. insulin), statistically significant improvements in glucose parameters relative to placebo were observed in the metformin stratum but not in the insulin stratum. Of note, the study was powered to detect differences between dapagliflozin and placebo for the overall population, not for individual strata. Patients in the insulin stratum tended to be older and have higher BMI, FPG, and HbA1c at baseline than patients in the metformin stratum. In addition, patients in the insulin stratum had longer disease duration. This, along with the fact that they were also treated with up to two OADs, suggests that they had more advanced disease and less residual islet function than those in the metformin group.

Differences between insulin and metformin strata may also reflect the expected greater effect of SGLT2 inhibitors on mealtime glucose excursions, most prominent in early diabetes. Patients in the metformin and insulin strata had similar estimated glomerular filtration rate values at baseline; thus, differential effects could not be attributed to a diminished effect of SGLT2 inhibitors because of worse kidney function in the insulin group. Although it is not known, patients in the metformin stratum may have additionally received a sulfonylurea that was discontinued before the lead-in period. A drift upward in fasting 24-h glucose is common when sulfonylureas are discontinued, and the lack of difference from baseline with dapagliflozin may be a reflection of this phenomenon, as evidenced by the deterioration seen among the placebo-treated patients.

The differences in outcomes between the metformin and insulin strata may also be a result of the study methodology. Patients in the insulin stratum were allowed to use rapid-acting mealtime insulin as part of the stable insulin dose regimen, and there was no run-in period to allow for restabilization of the daily insulin dose after initiating dapagliflozin. Any decrease in insulin dose, due to reductions in FPG upon adding dapagliflozin, may have affected efficacy measurements. Although no detailed insulin dosing information was collected, patients' adherence to the insulin regimen may have improved as part of their participation in a clinical trial, potentially contributing to reductions in MG.

Considered by many to be the gold standard for measuring glucose variability, MAGE was developed as a refinement in the characterization of glycemic instability and represents the arithmetic mean of the difference between consecutive peaks and nadirs exceeding 1 SD around the mean 24-h glucose value.^11,15,16^ The usefulness of MAGE is in quantifying major swings in glycemia while excluding minor swings. These major swings are thought to contribute to oxidative stress, which may play a key role in the pathogenesis of diabetic complications.^17^

The 24-h MG and MAGE results in this study corresponded with the increase in time spent in the target range, and the associated decrease in time spent in hyperglycemia (>180 mg/dL), with dapagliflozin versus placebo after 4 weeks of treatment. In the metformin stratum, this improvement in the glucose profile seemed most apparent overnight, suggestive of an increase in insulin sensitivity, which has been shown to exhibit diurnal changes.^18^

Although the addition of dapagliflozin increased the percentage of time spent with glucose <70 mg/dL relative to placebo (+1.3% vs. +0.3%, respectively), there were no severe hypoglycemic events. Few patients (dapagliflozin, *n* = 3; placebo, *n* = 3) experienced nonsevere symptomatic hypoglycemia, and all of these patients were in the insulin stratum. In clinical trials of dapagliflozin, mild hypoglycemia has been seen in ∼40% of those treated with dapagliflozin as an add-on to insulin with or without other OADs.^19^ The lower rate of hypoglycemia observed in this study with dapagliflozin plus insulin may be the result of the short duration of evaluation (4 weeks) versus other studies (24 weeks). The most common TEAE was urinary tract infection, which was balanced between the dapagliflozin and placebo groups (three patients each), and has been observed at a similar rate (≈6%) in other studies of similar populations.^19,20^

The effects of dapagliflozin on reducing glycemic variability in patients with T2D are in accordance with other CGM studies on the SGLT2 inhibitor class. In a 4-week randomized controlled study in patients with T2D (*n* = 60), empagliflozin significantly reduced 24-h MG and PPG, compared with placebo, and increased the percentage of time spent with glucose in the target range (≥70 to <180 mg/dL).^21^ The patient population consisted of Japanese patients who were drug naïve or treated with one OAD, which differed from this study in which patients were treated with either metformin monotherapy or insulin with or without up to two OADs, with results additionally analyzed in these strata.

Similarly, in an 8-week single-arm pilot study including patients with type 1 diabetes (*n* = 40), empagliflozin lowered glycemic variability and showed a trend toward increased time spent with glucose in the target range (≥70 to ≤140 mg/dL) compared with baseline, with more prominent effects on nighttime glycemia compared with daytime glycemia.^22^ Canagliflozin has also demonstrated improvements in indices of glycemic variability. In a substudy of an 18-week randomized controlled trial including patients with type 1 diabetes (*n* = 89), improvements versus placebo were observed in MG, glucose SD, and MAGE, with increased time spent with glucose in the target range (>70 to ≤180 mg/dL).^23^

In conclusion, dapagliflozin as an add-on therapy effectively reduced the glycemic measures of HbA1c, FPG, and PPG, consistent with several other trials performed as part of the dapagliflozin development program,^20,24^ and demonstrated the added benefit of stabilizing glucose concentrations for 24 h, regardless of meal intake or other elements of daily living. The results appear to be more robust with dapagliflozin plus metformin versus plus insulin, which may be an artifact of the study design, intrinsic to the combination regimen, or related to the patient population, with those on insulin having more advanced disease. Further investigations are needed to better characterize this relationship.

## Supplementary Material

Supplemental data

Supplemental data

Supplemental data

Supplemental data

Supplemental data

Supplemental data

Supplemental data

Supplemental data

Supplemental data

## References

[1] US Food and Drug Administration: Guidance for Industry. Diabetes Mellitus: Developing Drugs and Therapeutic Biologics for Treatment and Prevention. 2016 www.fda.gov/downloads/Drugs/GuidanceComplianceRegulatoryInformation/Guidances/ucm071624.pdf (accessed 818, 2016)

[2] McCarter RJ, Hempe JM, Chalew SA: Mean blood glucose and biological variation have greater influence on HbA1c levels than glucose instability: an analysis of data from the Diabetes Control and Complications Trial. Diabetes Care 2006;29:352–355 16443886 10.2337/diacare.29.02.06.dc05-1594

[3] Morimoto A, Nishimura R, Tsujino D, et al.: Relationship among A1C, hypoglycemia, and hyperglycemia in Japanese with type 2 diabetes—results from continuous glucose monitoring data. Diabetes Technol Ther 2011;13:667–670 21457068 10.1089/dia.2010.0230

[4] Pecoraro RE, Chen MS, Porte D Jr.: Glycosylated hemoglobin and fasting plasma glucose in the assessment of outpatient glycemic control in NIDDM. Diabetes Care 1982;5:592–599 6927729 10.2337/diacare.5.6.592

[5] New JP, Ajjan R, Pfeiffer AF, et al.: Continuous glucose monitoring in people with diabetes: the randomized controlled Glucose Level Awareness in Diabetes Study (GLADIS). Diabet Med 2015;32:609–617 25661981 10.1111/dme.12713

[6] Kim SK, Kim HJ, Kim T, et al.: Effectiveness of 3-day continuous glucose monitoring for improving glucose control in type 2 diabetic patients in clinical practice. Diabetes Metab J 2014;38:449–455 25541608 10.4093/dmj.2014.38.6.449 PMC4273031

[7] Pazos-Couselo M, Garcia-Lopez JM, Gonzalez-Rodriguez M, et al.: High incidence of hypoglycemia in stable insulin-treated type 2 diabetes mellitus: continuous glucose monitoring vs. self-monitored blood glucose. Observational prospective study. Can J Diabetes 2015;39:428–433 26254702 10.1016/j.jcjd.2015.05.007

[8] Kovatchev B, Cobelli C: Glucose variability: timing, risk analysis, and relationship to hypoglycemia in diabetes. Diabetes Care 2016;39:502–510 27208366 10.2337/dc15-2035 PMC4806774

[9] Komoroski B, Vachharajani N, Feng Y, et al.: Dapagliflozin, a novel, selective SGLT2 inhibitor, improved glycemic control over 2 weeks in patients with type 2 diabetes mellitus. Clin Pharmacol Ther 2009;85:513–519 19129749 10.1038/clpt.2008.250

[10] Parikh S, Wilding J, Jabbour S, et al.: Dapagliflozin in type 2 diabetes: effectiveness across the spectrum of disease and over time. Int J Clin Pract 2015;69:186–198 25438821 10.1111/ijcp.12531

[11] Service FJ, Molnar GD, Rosevear JW, et al.: Mean amplitude of glycemic excursions, a measure of diabetic instability. Diabetes 1970;19:644–655 5469118 10.2337/diab.19.9.644

[12] Marling CR, Shubrook JH, Vernier SJ, et al.: Characterizing blood glucose variability using new metrics with continuous glucose monitoring data. J Diabetes Sci Technol 2011;5:871–878 21880228 10.1177/193229681100500408 PMC3192592

[13] American Diabetes Association: 7: Approaches to glycemic treatment. Diabetes Care 2016;39:S52–S59 26696682 10.2337/dc16-S010

[14] Sharma M, Nazareth I, Petersen I: Trends in incidence, prevalence and prescribing in type 2 diabetes mellitus between 2000 and 2013 in primary care: a retrospective cohort study. BMJ Open 2016;6:e010210 10.1136/bmjopen-2015-010210 PMC4735176 26769791

[15] Monnier L, Colette C: Glycemic variability: should we and can we prevent it? Diabetes Care 2008;31:S150–S154 18227477 10.2337/dc08-s241

[16] Fritzsche G, Kohnert KD, Heinke P, et al.: The use of a computer program to calculate the mean amplitude of glycemic excursions. Diabetes Technol Ther 2011;13:319–325 21291337 10.1089/dia.2010.0108

[17] Monnier L, Mas E, Ginet C, et al.: Activation of oxidative stress by acute glucose fluctuations compared with sustained chronic hyperglycemia in patients with type 2 diabetes. JAMA 2006;295:1681–1687 16609090 10.1001/jama.295.14.1681

[18] Saad A, Dalla Man C, Nandy DK, et al.: Diurnal pattern to insulin secretion and insulin action in healthy individuals. Diabetes 2012;61:2691–2700 22751690 10.2337/db11-1478 PMC3478548

[19] AstraZeneca Pharmaceuticals LP: FARXIGA (dapagliflozin) tablets, for oral use [Prescribing Information]. 2016 www.azpicentral.com/farxiga/pi_farxiga.pdf (accessed 713, 2016)

[20] Wilding JP, Woo V, Soler NG, et al.: Long-term efficacy of dapagliflozin in patients with type 2 diabetes mellitus receiving high doses of insulin: a randomized trial. Ann Intern Med 2012;156:405–415 22431673 10.7326/0003-4819-156-6-201203200-00003

[21] Nishimura R, Tanaka Y, Koiwai K, et al.: Effect of empagliflozin monotherapy on postprandial glucose and 24-hour glucose variability in Japanese patients with type 2 diabetes mellitus: a randomized, double-blind, placebo-controlled, 4-week study. Cardiovasc Diabetol 2015;14:11 25633683 10.1186/s12933-014-0169-9 PMC4339254

[22] Perkins BA, Cherney DZ, Soleymanlou N, et al.: Diurnal glycemic patterns during an 8-week open-label proof-of-concept trial of empagliflozin in type 1 diabetes. PLoS One 2015;10:e0141085 26544192 10.1371/journal.pone.0141085 PMC4636141

[23] Rodbard HW, Peters AL, Slee A, et al.: The effect of canagliflozin, a sodium glucose cotransporter 2 inhibitor, on glycemic end points assessed by continuous glucose monitoring and patient-reported outcomes among people with type 1 diabetes. Diabetes Care 2017;40:171–180 27899497 10.2337/dc16-1353

[24] Bailey CJ, Gross JL, Pieters A, et al.: Effect of dapagliflozin in patients with type 2 diabetes who have inadequate glycaemic control with metformin: a randomised, double-blind, placebo-controlled trial. Lancet 2010;375:2223–2233 20609968 10.1016/S0140-6736(10)60407-2

