# Supplementary figures and images for: Effects of Dapagliflozin on 24-Hour Glycemic Control in Patients with Type 2 Diabetes: A Randomized Controlled Trial
Source: Diabetes Technol Ther. 2018 Oct 25;20(11):715–24. doi: 10.1089/dia.2018.0052 (PMC6208164; doi:10.1089/dia.2018.0052)

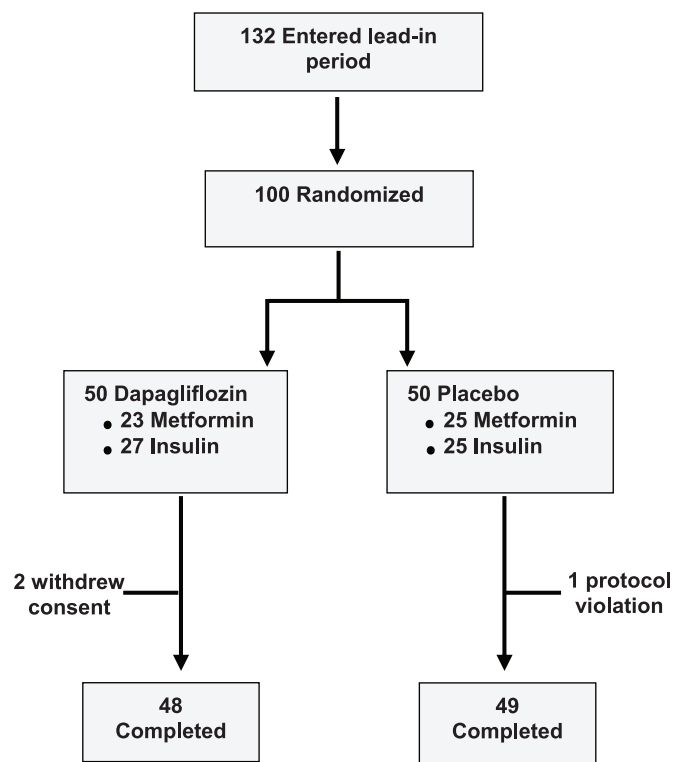

**SUPPLEMENTARY FIG. S2.** Study disposition.

Supplement: Supplemental data [file Supp_Fig2.pdf]
