# Supplementary material for: Effects of Dapagliflozin on 24-Hour Glycemic Control in Patients with Type 2 Diabetes: A Randomized Controlled Trial
Source: Diabetes Technol Ther. 2018 Oct 25;20(11):715–24. doi: 10.1089/dia.2018.0052 (PMC6208164; doi:10.1089/dia.2018.0052)
Supplement: Supplemental data [file Supp_Table1.pdf]

Supplementary Table S1. Antidiabetes Medications in the Insulin Stratum (N=52)

Medication class, n *(%)*	*N=52*
Long-acting insulin only	2 (3.9)
Long-acting insulin + OADs	13 (25.0)
Rapid-/short-acting insulin + long-/intermediate-acting insulin only	6 (11.5)
Rapid-/short-acting insulin + long-/intermediate-acting insulin + OADs	17 (32.7)
Other[a]	14 (26.9)

[a]Includes all patients who did not have any of the four treatment combinations mentioned.

OAD, oral antidiabetes drug.
