# Supplementary material for: Effects of Dapagliflozin on 24-Hour Glycemic Control in Patients with Type 2 Diabetes: A Randomized Controlled Trial
Source: Diabetes Technol Ther. 2018 Oct 25;20(11):715–24. doi: 10.1089/dia.2018.0052 (PMC6208164; doi:10.1089/dia.2018.0052)
Supplement: Supplemental data [file Supp_Fig3.pdf]

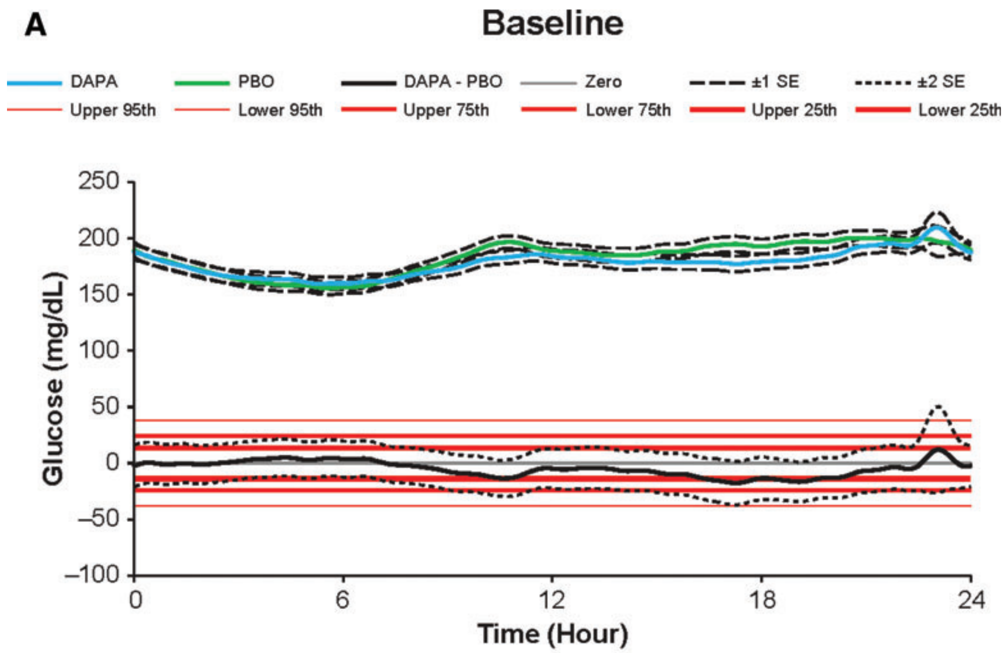

SUPPLEMENTARY FIG. S3. Comparison of mean 24-h glucose profiles at **(A)** baseline and **(B)** week 4, as shown by MADz in the overall population. Time 0 to 24 h means midnight to midnight. The black line represents the treatment DAPA–PBO difference; when the difference between the two groups (blue and green lines for DAPA and PBO, respectively) is outside the MADz red lines (95th percentiles), the two treatments are statistically different at that time of day. DAPA, dapagliflozin; MADz, maximum absolute deviation from zero; PBO, placebo; SE, standard error.
