# Supplementary material for: Effects of Dapagliflozin on 24-Hour Glycemic Control in Patients with Type 2 Diabetes: A Randomized Controlled Trial
Source: Diabetes Technol Ther. 2018 Oct 25;20(11):715–24. doi: 10.1089/dia.2018.0052 (PMC6208164; doi:10.1089/dia.2018.0052)
Supplement: Supplemental data [file Supp_Table2.pdf]

Supplementary Table S2. Time Spent in Plasma Glucose Ranges at Baseline and Week 4 in the Intention-to-Treat Population

	Overall (N = *100)*		*Metformin stratum* (n = *48)*		*Insulin stratum* (n = *52)*	
Characteristic	*Dapagliflozin* (n = *50)*	*Placebo* (n = *50)*	*Dapagliflozin* (n = *23)*	*Placebo* (n = *25)*	*Dapagliflozin* (n = *27)*	*Placebo* (n = *25)*
Time spent in BG >180 mg/dL, %						
Baseline, mean (SD)	40.9 (24.3)	44.1 (22.4)	40.2 (28.6)	45.5 (26.0)	41.4 (20.5)	42.6 (18.7)
Week 4, mean (SD)	29.1 (24.0)	46.8 (22.0)	24.9 (23.6)	52.9 (25.3)	32.5 (24.3)	41.0 (16.9)
Time spent in BG 70–180 mg/dL, %						
Baseline, mean (SD)	58.4 (24.1)	55.0 (22.2)	59.6 (28.6)	54.0 (25.3)	57.3 (20.0)	56.0 (19.1)
Week 4, mean (SD)	69.6 (23.7)	52.9 (21.9)	74.8 (23.6)	47.0 (25.2)	65.5 (23.4)	58.6 (16.8)
Time spent in BG <70 mg/dL, %						
Baseline, mean (SD)	0.8 (1.7)	1.0 (2.0)	0.2 (0.4)	0.5 (1.6)	1.3 (2.2)	1.4 (2.3)
Week 4, mean (SD)	1.3 (2.9)	0.3 (0.8)	0.4 (0.7)	0.1 (0.3)	2.0 (3.7)	0.4 (1.1)

BG, blood glucose; SD, standard deviation.
