# Supplementary material for: Effects of Dapagliflozin on 24-Hour Glycemic Control in Patients with Type 2 Diabetes: A Randomized Controlled Trial
Source: Diabetes Technol Ther. 2018 Oct 25;20(11):715–24. doi: 10.1089/dia.2018.0052 (PMC6208164; doi:10.1089/dia.2018.0052)
Supplement: Supplemental data [file Supp_Table3.pdf]

Supplementary Table S3. Key Secondary End Points

Secondary end points	Overall population		Metformin stratum		Insulin stratum	
	Dapagliflozin (n=50)	*Placebo* (n=50)	*Dapagliflozin* (n=23)	*Placebo* (n=25)	*Dapagliflozin* (n=27)	*Placebo* (n=25)
Fructosamine, mg/dL						
Baseline mean (SD)	293.5 (6.7)	307.9 (7.3)	279.3 (8.9)	310.0 (11.5)	305.5 (9.4)	305.8 (9.3)
Adjusted mean (SE) change from baseline to week 4	−20.4 (3.2)	−9.6 (3.2)	−20.2 (4.9)	−2.2 (4.6)	−21.8 (4.3)	−16.3 (4.4)
Adjusted mean (SE) difference vs. placebo	−10.8 (4.6)		−18.1 (6.9)		−5.5 (6.1)	
P value for treatment difference	0.019		0.012		0.373	
24-h MAGE, mg/dL						
Baseline mean (SD)	102.7 (31.0)	108.6 (29.9)	89.3 (24.8)	98.9 (22.6)	114.0 (31.5)	118.2 (33.4)
Adjusted mean (SE) change from baseline to week 4	−10.0 (4.1)	+5.3 (4.1)	−7.3 (6.2)	+10.4 (5.8)	−12.7 (5.5)	+0.2 (5.7)
Adjusted mean (SE) difference vs. placebo	−15.3 (5.8)		−17.7 (8.5)		−12.9 (7.9)	
P value for treatment difference	0.010		0.040		0.105	
''Distance traveled,'' mg/dL						
Baseline mean (SD)	793.9 (267.5)	779.7 (167.0)	781.9 (338.9)	749.7 (184.8)	804.2 (193.6)	809.7 (144.6)
Adjusted mean (SE) change from baseline to week 4	−28.0 (26.3)	+9.5 (25.9)	−50.0 (39.5)	+54.1 (36.9)	−5.9 (34.8)	−35.0 (36.2)
Adjusted mean (SE) difference vs. placebo	−37.5 (36.9)		−104.1 (54.1)		+29.1 (50.2)	
P value for treatment difference	0.312		0.057		0.564	
SD of 24-h glucose, mg/dL						
Baseline mean (SD)	42.9 (13.1)	43.8 (11.9)	36.7 (10.7)	39.0 (8.9)	48.1 (12.9)	48.5 (12.7)
Adjusted mean (SE) change from baseline to week 4	−3.4 (1.6)	+1.3 (1.6)	−1.6 (2.4)	+5.1 (2.2)	−5.1 (2.1)	−2.5 (2.2)
Adjusted mean (SE) difference vs. placebo	−4.7 (2.2)		−6.8 (3.3)		−2.7 (3.0)	
P value for treatment difference	0.037		0.041		0.382	

Study was powered only for the overall population; although *P* values are supplied for the individual strata, inferences for treatment differences should not be made.
MAGE, mean amplitude of glucose excursion; SE, standard error.
