# Supplementary material for: Effects of Dapagliflozin on 24-Hour Glycemic Control in Patients with Type 2 Diabetes: A Randomized Controlled Trial
Source: Diabetes Technol Ther. 2018 Oct 25;20(11):715–24. doi: 10.1089/dia.2018.0052 (PMC6208164; doi:10.1089/dia.2018.0052)
Supplement: Supplemental data [file Supp_Table4.pdf]

Supplementary Table S4. Summary of Treatment-Emergent Adverse Events by System Organ Class and Preferred Term: Safety Population

System organ class preferred term, n *(%)*	*Dapagliflozin* (n=*50*)	*Placebo* (n=*50*)	*Overall* (N=*100*)
Patients with any TEAE	18 (36)	10 (20)	28 (28)
Infections and infestations	7 (14)	5 (10)	12 (12)
Urinary tract infection	3 (6)	3 (6)	6 (6)
Upper respiratory tract infection	1 (2)	2 (4)	3 (3)
Genital infection, fungal	1 (2)	0	1 (1)
Nasopharyngitis	1 (2)	0	1 (1)
Tooth abscess	1 (2)	0	1 (1)
Gastrointestinal disorders	4 (8)	0	4 (4)
Abdominal discomfort	1 (2)	0	1 (1)
Abdominal distention	1 (2)	0	1 (1)
Diarrhea	1 (2)	0	1 (1)
Flatulence	1 (2)	0	1 (1)
Nausea	1 (2)	0	1 (1)
Vomiting	1 (2)	0	1 (1)
Renal and urinary disorders	3 (6)	1 (2)	4 (4)
Glycosuria	1 (2)	0	1 (1)
Hematuria	1 (2)	0	1 (1)
Nephrolithiasis	0	1 (2)	1 (1)
Pollakiuria	1 (2)	0	1 (1)
Skin and subcutaneous tissue disorders	2 (4)	1 (2)	3 (3)
Dermatitis, contact	1 (2)	0	1 (1)
Rash	0	1 (2)	1 (1)
Rash, papular	1 (2)	0	1 (1)
Blood and lymphatic system disorders	1 (2)	1 (2)	2 (2)
Anemia	1 (2)	1 (2)	2 (2)
General disorders and administration-site conditions	0	2 (4)	2 (2)
Application-site irritation	0	1 (2)	1 (1)
Influenza-like illness	0	1 (2)	1 (1)
Injury, poisoning, and procedural complications	0	2 (4)	2 (2)
Animal bite	0	1 (2)	1 (1)
Muscle strain	0	1 (2)	1 (1)
Investigations	2 (4)	0	2 (2)
Blood creatinine increased	1 (2)	0	1 (1)
GFR decreased	1 (2)	0	1 (1)
White blood cells, urine positive	1 (2)	0	1 (1)
Respiratory, thoracic, and mediastinal disorders	2 (4)	0	2 (2)
Oropharyngeal pain	2 (4)	0	2 (2)
Metabolism and nutrition disorders	1 (2)	0	1 (1)
Decreased appetite	1 (2)	0	1 (1)
Musculoskeletal and connective tissue disorders	0	1 (2)	1 (1)
Pain in extremity	0	1 (2)	1 (1)
Nervous system disorders	1 (2)	0	1 (1)
Headache	1 (2)	0	1 (1)
Vascular disorders	1 (2)	0	1 (1)
Hypotension	1 (2)	0	1 (1)

Adverse events were coded by Medical Dictionary for Regulatory Activities version 17.1. Primary system organ classes were sorted by total incidences (descending); preferred terms were sorted within primary system organ class by total incidences (descending).

Safety population consisted of all patients who received at least one dose of investigational product, analyzed according to the type of investigational product received.

GFR, glomerular filtration rate; TEAE, treatment-emergent adverse event.
